# The effect of infant vitamin B_12_ supplementation on neurodevelopment: a follow-up of a randomised placebo-controlled trial in Nepal

**DOI:** 10.1017/S0007114522000071

**Published:** 2023-01-14

**Authors:** Manjeswori Ulak, Ingrid Kvestad, Ram Krishna Chandyo, Suman Ranjitkar, Mari Hysing, Catherine Schwinger, Merina Shrestha, Sudha Basnet, Laxman P. Shrestha, Tor A. Strand

**Affiliations:** 1 Centre for Intervention Science in Maternal and Child Health, Centre for International Health, Department of Global Public Health and Primary Care, University of Bergen, Bergen, Norway; 2 Department of Child Health, Institute of Medicine, Tribhuvan University, Kathmandu, Nepal; 3 Regional Centre for Child and Youth Mental Health and Child Welfare, NORCE Norwegian Research Centre, Bergen, Norway; 4 Department of Research, Innlandet Hospital Trust, Lillehammer, Norway; 5 Department of Community Medicine, Kathmandu Medical College, Kathmandu, Nepal; 6 Department of Psychosocial Science, Faculty of Psychology, University of Bergen, Bergen, Norway

**Keywords:** Vitamin B_12_ supplementation, Full scale IQ, Cognition, Neurodevelopment, Children, Nepal

## Abstract

The most critical period for brain development is before a child’s second birthday. Standardised tests measuring neurodevelopment are more reliable when administered after this period. Severe vitamin B_12_ deficiency affects brain development and function. In a randomised, double-blind, placebo-controlled trial in 600 Nepalese infants (6–11 months at enrolment), we found no effect of 2 µg vitamin B_12_ daily for a year on neurodevelopment. The primary objective of the current study was to measure the effect of the intervention on the Wechsler Preschool and Primary Scale of Intelligence (WPPSI-IV) full scale intelligence quotient (FSIQ). We measured the effect on the Bayley Scales of Infant and Toddler Development 3rd edition at age 30–35 months (*n* 555). At age 42–47 months (*n* 533), we used the WPPSI-IV and subtests from the Neuropsychological Assessment, 2nd edition (NEPSY-II). We also used the FSIQ to estimate subgroup specific effects. The mean (sd) WPPSI-IV FSIQ in the vitamin B_12_ group was 84·4 (8·4) and 85·0 (8·6) in the placebo group (mean difference −0·5 (95 % CI -1·97, 0·94), *P* = 0·48). There were no effect of the vitamin B_12_ on any of the other neurodevelopmental outcomes and no beneficial effect in any of the subgroups. In conclusion, providing 2 µg of vitamin B_12_ for a year in infants at risk of vitamin B_12_ deficiency does not improve preschool cognitive function.

Vitamin B_12_ (cobalamin) is essential for brain development, and deficiency is common in many low- and middle-income countries^(1)^. Vitamin B_12_ status depends largely on the amount of animal source foods in the diet^(2)^, and in South Asian countries, where diets are predominantly vegetarian, poor vitamin B_12_ status is common^(3,4,5)^. Breastfed infants have a higher risk of poor vitamin B_12_ indices than weaned infants^(3,4)^. In Nepal, consumption of animal products particularly among children is very low^(6)^.

Vitamin B_12_ is required for cell division and differentiation, energy production and the generation of methionine, which is needed to produce neurotransmitters and myelin in the brain^(7,8)^. Subclinical vitamin B_12_ deficiency is associated with poor cognitive performance in both the elderly and children^(1,9,10)^. Results from an Indian cohort study suggested that children with inadequate vitamin B_12_ status had lower scores on the Bayley Scales of Infant and Toddler Development 2nd edition (Bayley-II) than replete children^(9)^. Moreover, in a cohort of Nepalese children, we found that vitamin B_12_ status in infancy was positively associated with cognitive function when the children were 5 years old^(11)^. In two randomised-controlled trials (RCT) in Norwegian infants with signs and symptoms of vitamin B_12_ deficiency, a high dose of vitamin B_12_ improved short-term motor functioning^(12,13)^. Another RCT in North India reported a small borderline significant beneficial effect of 1·8 µg vitamin B_12_ supplementation given daily for 6 months on neurodevelopment in young children, with the most pronounced effect among children who had evidence of vitamin B_12_ deficiency and who were stunted at study start^(14)^.

Whether the results from these aforementioned RCTs can be generalised to populations without overt vitamin B_12_ deficiency is not known. We recently undertook an RCT in mildly stunted Nepalese infants receiving 2 µg of vitamin B_12_ or placebo for one year starting when they were 6–11 months old^(15)^. This amount of vitamin B_12_ corresponds to approximately 2–3 recommended daily allowances depending on the age of the child^(16)^. We did not find any effect of vitamin B_12_ supplmentation on growth or neurodevelopment after one year of supplementation despite an excellent compliance to the supplements and a metabolic response reflecting improved vitamin B_12_ status in the intervention group^(15)^. The reliability of neurodevelopmental measures during the first years of life is limited because children develop rapidly and at an uneven pace during this period^(17)^. This may affect the ability to detect differences between groups in young children. The reliability of the tools improves with age^(18)^ and measures taken when the children are older could accordingly be better suited to identify changes to the developing brain that started during infancy. The objective of the current study was to measure the effects of vitamin B_12_ supplementation that started in infancy on neurodevelopment at 30–47 months of age, with the Wechsler Preschool and Primary Scale of Intelligence 4th edition (WPPSI-IV) full scale intelligence quotient (FSIQ) at 42–47 months as the primary outcome.

## Subjects and methods

### Study setting and participants

The study is a follow-up of a recently completed double-blind randomised placebo-controlled trial conducted in Bhaktapur municipality, Nepal and surrounding areas^(15)^. Enrollment in the original study took place from April 2015 to February 2018 where 600 children aged 6 to 11 months with a length-for-age < −1 *Z*-score and who planned to reside in the field site at least for the next 12 months were included^(15)^. Children were excluded in case of acute or chronic illness, severe malnutrition (weight-for-length *Z*-score <-3), severe anaemia (Hb<7 g/dl), if they were taking multivitamin supplements containing vitamin B_12_, if they required treatment with vitamin B_12_ or if parents or caretakers did not consent to participate in the study. Details on study protocol and the random selection procedure for the study have been published elsewhere^(15)^. The assignment to intervention groups was concealed and blinded to all study personnel and participants. The intervention consisted of daily administration of a paste containing either 2 µg (2–3 recommended daily allowances) of vitamin B_12_ (cyancobalamin) or a placebo over a period of 12 months. The supplement was delivered in sachets (eeZee20) containing 20 g of a lipid-based, 108 kcal nutrient paste produced by GC Rieber Compact (http://www.gcriebercompact.com). The sachets contained either vitamin B_12_ or placebo, and in addition an equal quantity of other multivitamins and minerals at approximately 1 recommended daily allowance. The main outcomes of the trial were growth, neurodevelopment and Hb concentration measured after 12 months of supplementation^(15)^.

Between April 2017 and February 2020, we approached all participating children who had not dropped out of the main study (*n* 574), 1 and 2 years after the end of supplementation (i.e., children aged 30–35 months and 42–47 months, respectively) for follow-up assessments. Field workers contacted the families either via phone or through a visit to their homes. We also informed families who had moved outside of the area about the extended follow-up and requested them to visit the study clinic according to the scheduled activities. On the days of assessment, the study psychologist obtained written informed consent from the children’s caregiver for the purpose of the present follow-up study.

### Data collection

Trained field workers collected information on the family’s demographics and socio-economic situation, as well as the child’s morbidity history at enrollment. They also measured length and weight and collected blood samples. Weight was measured to the nearest 50 g using Seca scales. Length was measured by wooden infantometer (UNICEF), reading to the nearest 0·1 cm. Blood was collected up to 4 ml into a polypropylene tube containing EDTA (Sarstedt, Germany). Immediately after the blood sampling, Hb was measured by Hemocue (Ångelholm, Sweden), and plasma was separated by centrifugation at room temperature (for 10 min at 700 *g*), transferred into storage vials and stored at < -80°C until analysis. The plasma concentrations of vitamin B_12_ and the metabolic markers of total homocysteine and methylmalonic acid (MMA) were analysed at Bevital laboratory, Bergen, Norway. We also calculated a combined indicator of vitamin B_12_ status (3cb12) based on the three biomarkers vitamin B_12_, total homocysteine and MMA. For the follow-ups, field workers completed a questionnaire concerning morbidity, schooling and growth of the participant children and the study psychologists performed neurodevelopmental assessments.

### Neurodevelopmental outcomes

In the first follow-up, when the children were 30–35 months old, they were assessed with the Bayley-III. In the second follow-up at 42–47 months, children were assessed using the WPPSI-IV and the Neuropsychological Assessment, 2nd edition (NEPSY-II). The neurodevelopmental assessments for both follow-ups were conducted at the study clinic in well-lit rooms with minimal distractions. The children were accompanied by their caregivers who had the opportunity to sit in the back of the room during the assessments. Three study psychologists performed all assessments and quality control. Standardisation exercises were performed in twenty children per tester for each test. Double scoring was done throughout the study in 10 % of all assessments by two psychologists. We attained an inter-correlation coefficient >98 % for both standardisation and double scoring indicating excellent inter-rater agreement.

Bayley-III is a standard assessment tool of developmental functioning in infants and toddlers aged 1–42 months. It is one of the most widely used tools worldwide for research purposes in this age group^(19)^. The Bayley-III consists of a series of developmental tasks and takes between 45 and 60 min to administer. For the current study, we used both the four subscale composite scores (i.e. Cognitive, Language, Motor and Socio-emotional) and the six subtest scaled scores (i.e. Cognitive, Receptive and Expressive language, Fine and Gross motor and Socio-emotional). The raw scores of each subscales were converted into scaled and composite scores based on American norms^(19)^. The Bayley-III scaled scores have an expected mean (standard deviation (sd)) of 10 (3), and the composite scores have a expected mean (sd) of 100 (15)^(19)^. The Bayley-III scales was adapted and translated for the Nepalese context, and its feasibility and reliability for use in Nepalese children have been discussed previously^(20,21)^. Translation of the test instructions was done according to standard procedures, and small adaptions to the materials (i.e. changing pictures and drawings in the picture and stimulus books) were done to improve the acceptability for the Nepalese setting.

WPPSI-IV is a clinical assessment tool of intellectual ability in children aged 2 years and 6 months through 7 years and 7 months (2:6 to 7:7)^(22)^. We used six subtests; Receptive vocabulary, Block Design, Picture Memory, Object Assembly, Zoo Locations and Information to generate standardised composite scores for the FSIQ. In addition, we used three indexes: Verbal Comprehension Index, Visual Spatial Index and Working Memory Index. The raw scores of each subscales were converted into index scores and FSIQ scores with an expected mean (sd) of 100 (15). The calculation of the FSIQ scores was based on American norms, as there were no Nepalese norms^(19)^. The WPPSI-IV takes 45–60 min to administer^(23)^.

NEPSY-II is a neuropsychological cognitive assessment tool that can be tailored for children aged 3–16 years^(24)^. The NEPSY-II consists of thirty-two subtests in six functional domains. The following three age appropriate subtests were administered in this study: Affect Recognition, Statue and Geometric Puzzles from the Social Perception, Executive Functioning and Visual-Spatial Processing domains, respectively. The subscale raw scores were converted into scaled scores for the subtest by age group based on American norms. For the geometric puzzles, there were no norms for this age range, so we used raw scores^(25)^. The NEPSY-II scaled scores have a mean (sd) of 10 (3). After careful consideration of the test material, no changes were made to the WPPSI and NEPSY test for the current study.

### Sample size

Of the original sample of 600, we were able to measure neurodevelopment in at least 533 children for both of the time points during the extended follow-up. This follow-up rate ensured a statistical power of 82 and 93 % to detect minimal meaningful differences of 0·25 and 0·3 standardised effect sizes, respectively. These calculations assumed equal variances between the groups and an *α* of 0·05. For details regarding the sample size estimations, please see the protocol paper^(26)^.

### Ethics

The study obtained approval from the Nepal Health Research Council (Reg.no.73/2017) in Nepal and from REC Norway (2014/1528). The clinical trials register number is NCT02272842.

### Statistical analysis

All forms were manually checked for inconsistencies by field supervisors, and data were entered in computerised databases; discrepancies and completeness were checked by the data entry supervisor. Analyses were performed using Stata® version 16 (Stata Corp.)^(27)^. Proportions and mean (sd) for all baseline categorical and continuous variables were calculated for each intervention group. Children’s weight-for-age, weight-for-length/height and length/height-for-age *Z*-score were calculated based on the WHO growth standards^(28)^. Vitamin B_12_ status at baseline and at the end of the study was described in the manuscript reporting the main outcomes^(15,29)^. Means (sd) and mean differences (95 % CI) for the cognitive outcomes between intervention groups were calculated. In linear regression analyses, we examined the effect of the intervention on the primary outcome (WPPSI-IV FSIQ) in pre-defined subgroups, which were the same as reported in the main paper^(26)^. These subgroups were defined according to baseline vitamin B_12_ status (i.e., plasma concentration of vitamin B_12_, total homocysteine, MMA and 3cb12), as well as according to baseline anaemia status, birth weight, stunting, wasting and underweight. The subgroup effects are presented in forest plots (metan function in Stata® version 16).

## Results

**Fig. 1.**
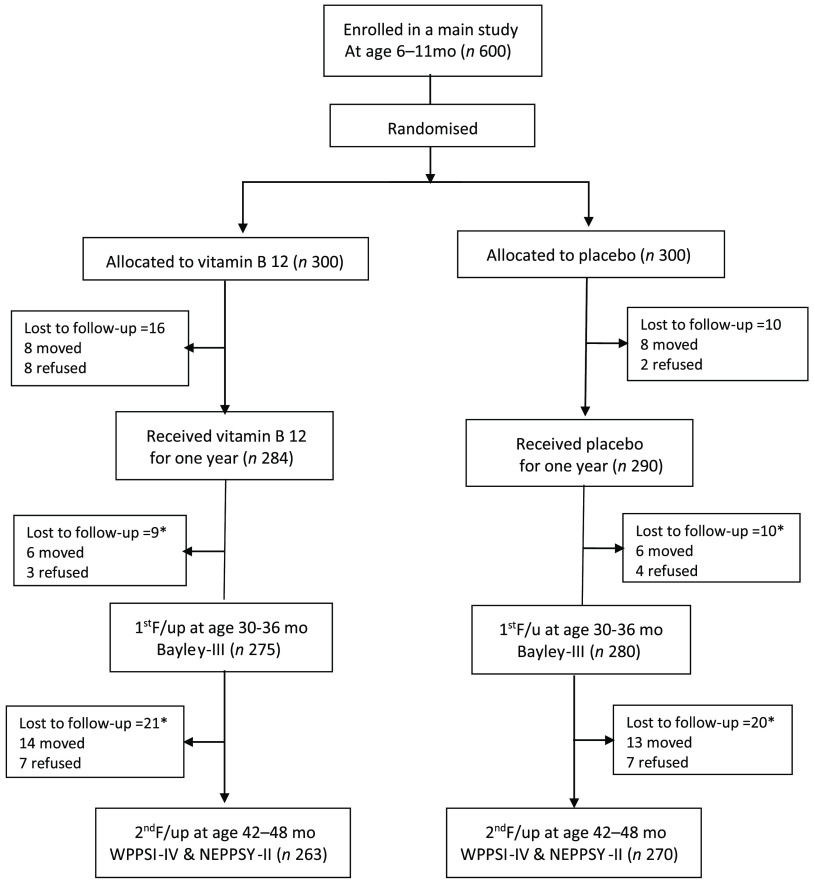
Trial profile of a randomised control trial on vitamin B_12_ in Nepalese infants and a follow-up study 1 and 2 years after end of the intervention. *Number of lost to follow-up of the 1st and 2nd /follow-ups were from the total number of 574 participants who completed the one year supplementation.

Among 600 participant infants, we were unable to follow-up on twenty-six participants from the original study mainly due to migration or refusal to participate. Among the 574 children who completed the intervention, we were able to re-consent 555 (96·7 %) at the first scheduled time point and 533 (92·8 %) at the second scheduled time point. The main reason for not being re-enrolled in the follow-up rounds was migration (Fig. 1).

Demographic characteristics among the intervention and placebo group at enrollment were similar (Table 1). The mean age of the children at enrollment was 8 months (sd 1·8). Almost one-half of the participants lived in joint families and half of the participants in rented houses. One in every five children was born with low birth weight and one-third were stunted at enrollment (LAZ < -2 *Z* score).

**Table 1.** Baseline information of the 6–11-month-old Nepalese infants in the original randomised-controlled trail (Numbers and percentages; mean values and standard deviations, *n* 600)

	Vitamin B_12_ group (*n* 300)		Placebo group (*n* 300)	
	Mean/*n*	sd/%	Mean/*n*	sd/%
Infant characteristics				
Age of child (months)	8·1	1·7	8·0	1·8
Male child	158	53	151	50
Have older siblings	155	52	153	51
Low birth weight (<2500 g)*	56	19	59	20
Hospitalisation within 1st month of age	28	9·3	26	8·7
Demographic features				
Mother’s age	27·1	4·7	27·5	4·6
Father’s age^†^	30·0	7·1	30·6	5·1
Mothers who completed secondary school or above	197	65·7	180	60
Fathers who completed secondary school or above	199	66·3	189	63
Mothers who work	117	39·0	110	36·7
Fathers who work†	286	95·3	280	93·3
Socio-economic status				
Family staying in joint family	143	47·7	149	49·7
Family residing in rented house	152	50·7	139	46·3
Number of rooms in use by the household (≤2)	163	54·3	174	58
Kitchen and bedroom in same room	148	49·3	150	50
Family having own land	138	46	144	48
Receiving remittance from abroad	30	10	27	9
Breast-feeding status				
No breast-feeding at time of interview	8	2·7	6	2
Exclusive breast-feeding for 3 months or more	143	47·7	137	45·6
Nutritional status of infants				
Underweight (weight for age *Z*-score <2)	62	20·7	50	16·6
Stunting (length for age *Z*-score <2)	96	32·1	98	32·7
Wasting (weight for length *Z*-score <2)	12	4	7	2·3
Hb, g/dl	10·6	0·96	10·6	0·91
Anemia (Hb <11 g/dl)	183	61	202	67·3
Nutritional status of mother				
BMI of mother	23·7	3·5	23·7	3·6
<18·5 kg/m^2^ BMI of mother	15	5	19	6·3

*n*, number.

* Among 579 infants from whom birth weight was recorded.

† Among 487 fathers who were available.

Means (sd) and the mean differences of the cognitive outcomes by intervention group are shown in Table 2. For the primary outcome (i.e., WPPSI-IV FSIQ), the mean (sd) score for the vitamin B12 and placebo group were 84·4 (8·4) and 85·0 (8·6), respectively, with a mean difference of −0·5 (95 % CI -1·97, 0·94). Likewise, there were no significant differences between the intervention groups in any of the other study outcomes.

**Table 2.** Mean (sd) neurodevelopmental scores by study group at the follow-ups in Nepalese children aged 30 to 47 months old in Bhaktapur (Numbers and percentages; mean values and standard deviations)

	Vitamin B_12_ group		Placebo group		Mean difference	95 % CI	*P*-value
	Mean	sd	Mean	sd			
WPPSI-IV subscales, age 42–47 mo	*n* 269		*n* 264				
Full scale IQ*	84·4	8·4	85·0	8·6	–0·5	–1·97, 0·94	0·48
Verbal Comprehension Index score	83·7	8·0	84·2	7·7	–0·4	–1·79, 0·89	0·51
Visual Spatial Index score	85·3	8·3	85·8	8·4	–0·5	–1·95, 0·91	0·47
Working Memory Index score	103·0	13·2	104·0	13·4	–0·9	–3·24, 1·28	0·39
NEPSY-II subscales, age 42–47 mo							
Affect recognition scaled score†	8·2	2·0	8·0	1·8	0·1	–0·13, 0·53	0·24
Geometric puzzles raw score	9·1	2·6	9·0	2·6	0·1	–0·36, 0·59	0·63
Statue scaled score	10·2	3·1	10·1	3·3	0·1	–0·44, 0·66	0·70
Bayley-III subscales, age 30–35 mo	*n* 281		*n* 274				
Cognitive composite score‡	84·9	6·9	85·5	6·9	–0·5	–1·72, 0·59	0·34
Motor composite score	103·7	9·9	104·4	9·8	–0·7	–2·38, 0·92	0·38
Gross motor scaled score	9·5	1·8	9·5	1·6	–0·0	–0·34, 0·23	0·71
Fine motor scaled score	11·6	2·3	11·8	2·4	–0·1	–0·58, 0·21	0·36
Language composite score	95·0	7·5	95·0	7·4	–0·0	–1·30, 1·20	0·93
Language receptive scaled score	9·1	1·2	9·1	1·2	–0·0	–0·24, 0·18	0·78
Language expressive scaled score	9·1	1·2	9·1	1·2	0·1	–0·27, 0·29	0·93
Socio-emotional composite score	90·1	12·9	89·6	12·7	0·5	–1·57, 2·69	0·60

WPPSI-IV, Wechsler Preschool and Primary Scale of Intelligence fourth edition; NEPSY-II, Neuropsychological Assessment, 2nd edition; Bayley-III, Bayley Scales of Infant and Toddler Development, 3rd edition.

* Mean (sd) of FSIQ and index scores 100 (15).

† Mean (sd) of scaled score 10 (3).

‡ Mean (sd) of composite and Index score 100 (15), the scaled scores and composite scores were calculated based on American norms.

**Fig. 2.**
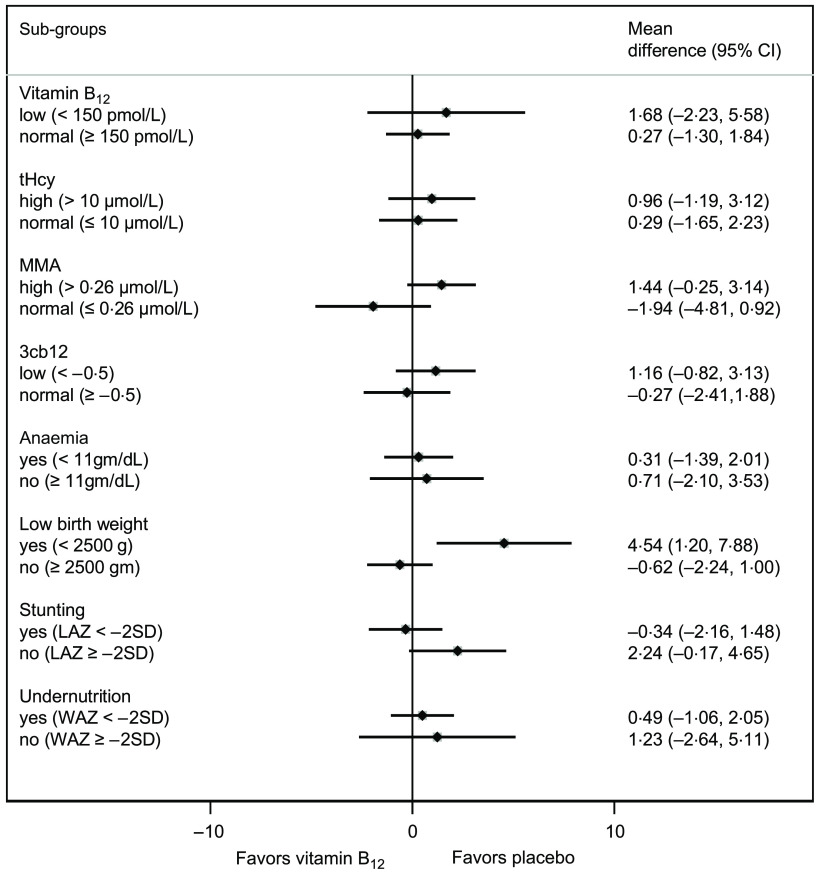
The effect of vitamin B_12_ supplementation in infancy in pre-defined subgroups on the Wechsler Preschool and Primary Scale of Intelligence (WPPSI-IV), Full Scale Intelligence Quotient. MMA, methylmalonic acid; tHcy, total homocysteine; 3cb12, combined indicator of vitamin B_12_ status, calculated based on vitamin B_12_, MMA and tHcy; LAZ, length for age, WAZ, weight for age; *P*-value for interaction between birthweight and intervention group: 0·002; all other *P*-values for interaction are not significant (>0·05).

The effects in the different subgroups are shown in Fig. 2. There were no significant differences between the intervention groups in any of the subgroups on the WPPSI-IV FSIQ, except for those born at low birth weight. In this subgroup, vitamin B_12_ supplementation leads to 4·54 (95 % CI 1·20, 7·88) lower FSIQ score. The interaction between intervention group and birth weight was statistically significant (interaction term regression coefficient: 4·02 (95 % CI 1·52, 6·51), *P* = 0·002).

## Discussion

In this follow-up study, our objective was to examine potential changes to the developing brain from 1 year vitamin B_12_ supplementation during infancy, on neurodevelopment measured beyond 30 months of child age when these measures are considered more reliable. We were not able to demonstrate any effect from the 1 year vitamin B_12_ supplementation on neurodevelopment at this age overall or in pre-defined sub-groups.

To our knowledge, this is one of the first studies that have examined the long-term relationship between vitamin B_12_ supplementation in infancy and neurodevelopmental outcomes after 3 years of age. Our findings are similar to the results from a long-term follow-up of a RCT in India, where 6 months of vitamin B_12_ and/or folic acid supplementation in early childhood was not associated with cognitive outcomes when the children reached school age^(30)^. Notably, in this RCT, the dose of vitamin B_12_ was 2 to 3 recommended daily allowances, similar to the current trial. Our findings in this follow-up are also in accordance with the results from the original trial where there was no effect of vitamin B_12_ supplementation on neurodevelopment measured with Bayley-III immediately following the end of supplementation when the children were 18–23 months old^(15)^. The lack of effect was in spite of an excellent compliance to the supplements, and a clear metabolic response reflecting improved vitamin B_12_ status. The aim of the current study was to examine potential long-term effects of the vitamin B_12_ intervention at ages when neurodevelopmental measures are considered to be more reliable. Given the null findings of the current follow-up, the results substantiate the fact that providing 2 µg vitamin B_12_ daily to children during infancy does not benefit neurodevelopment in children.

Our finding contradicts two Norwegian RCT where improved motor development was seen among mild vitamin B_12_-deficient infants following a high-dose vitamin B_12_ provided intramuscularly^(12,13)^. The contrasting findings could be due to poor absorption and/or the relatively low dose in the current study, and a higher dose of vitamin B_12_ provided intramuscularly could provide a more favourable impact on the neurodevelopmental outcomes. Moreover, although we targeted marginally stunted infants at enrollment with the presumption of prevalent vitamin B_12_ deficiency among these children^(5)^, only 11 % of the infants were vitamin B_12_ deficient (<148 pmol/l) and <2 % of the families were practicing a vegetarian diet. The low occurrence of vitamin B_12_ deficiency could be another explanation for the lack of an effect of the supplementation.

In the subgroup analyses, a surprising finding was a negative effect of vitamin B_12_ on WPPSI-IV FSIQ seen in low birth weight children. To the best of our knowledge, there are no previous reports of any adverse effect of vitamin B_12_ in low birth weight children. This is one out of many subgroup analyses and may be a chance finding. However, this apparent negative effect of vitamin B_12_ in low birth weight babies, needs to be explored in further studies.

Strengths of the study include the fact that we were able to re-enroll >93 % of the original study children after 2 years of the end of supplementation and the use of high quality, comprehensive and validated tools such as the Bayley-III, WIPPSI-IV and NEPSY-II when children were 3 years and above. There are also a few limitations of the study. We gave a peanut-based paste of 20 mg containing either 2 µg of vitamin B_12_ or placebo. Field workers did weekly visits, and we observed excellent compliance to the supplementation. However, in case of difficulties of feeding the peanut-based paste, social desirability might have led to over-reporting by the mothers. The neurodevelopmental tools were not validated for a Nepali context and as no local norms existed, we have used American norms when converting from raw scores to subscale and composite scores.

### Conclusion

We found no long-term effect of vitamin B_12_ supplementation during early childhood on neurodevelopment in children at age 30–47 months. Our findings do not support supplementation of vitamin B_12_ around the recommended daily intake in infants from low- and middle-income countries to improve brain development. Further studies are needed to investigate the importance of vitamin B_12_ for childhood neurodevelopment.

## Supplementary material

For supplementary material accompanying this paper visit https://doi.org/S0007114522000071. click here to view supplementary material

## References

[1] Black MM (2008) Effects of vitamin B_12_ and folate deficiency on brain development in children. Food Nutr Bull 29, S126–S131. 18709887 10.1177/15648265080292S117 PMC3137939

[2] Murphy SP & Allen LH (2003) Nutritional importance of animal source foods. J Nutr 133, 3932S–3935S. 14672292 10.1093/jn/133.11.3932S

[3] Taneja S , Bhandari N , Strand TA , et al. (2007) Cobalamin and folate status in infants and young children in a low-to-middle income community in India. Am J Clin Nutr 86, 1302–1309. 17991639 10.1093/ajcn/86.5.1302

[4] Ulak M , Chandyo RK , Adhikari RK , et al. (2014) Cobalamin and folate status in 6 to 35 months old children presenting with acute diarrhea in Bhaktapur, Nepal. PLOS ONE 9, e90079. 24594935 10.1371/journal.pone.0090079 PMC3940712

[5] Ulak M , Chandyo RK , Thorne-Lyman AL , et al. (2016) Vitamin status among breastfed infants in Bhaktapur, Nepal. Nutrients 8, 149. 27005657 10.3390/nu8030149 PMC4808878

[6] Shrestha L , Parmar A , Kulig B , et al. (2020) Feeding practices of pre-school children and associated factors in Kathmandu, Nepal. J Hum Nutr Diet 33, 241–251. 31680361 10.1111/jhn.12715

[7] Dror DK & Allen LH (2008) Effect of vitamin B_12_ deficiency on neurodevelopment in infants: current knowledge and possible mechanisms. Nutr Rev 66, 250–255. 18454811 10.1111/j.1753-4887.2008.00031.x

[8] Finkelstein JL , Layden AJ & Stover PJ (2015) Vitamin B_12_ and perinatal health. Adv Nutr 6, 552–563. 26374177 10.3945/an.115.008201 PMC4561829

[9] Strand TA , Taneja S , Ueland PM , et al. (2013) Cobalamin and folate status predicts mental development scores in North Indian children 12–18 months of age. Am J Clin Nutr 97, 310–317. 23283502 10.3945/ajcn.111.032268

[10] van de Rest O , van Hooijdonk LW , Doets E , et al. (2012) B vitamins and *n*-3 fatty acids for brain development and function: review of human studies. Ann Nutr Metab 60, 272–292. 22678093 10.1159/000337945

[11] Kvestad I , Hysing M , Shrestha M , et al. (2017) Vitamin B_12_ status in infancy is positively associated with development and cognitive functioning 5 years later in Nepalese children. Am J Clin Nutr 105, 1122–1131. 28330909 10.3945/ajcn.116.144931

[12] Torsvik I , Ueland PM , Markestad T , et al. (2013) Cobalamin supplementation improves motor development and regurgitations in infants: results from a randomized intervention study. Am J Clin Nutr 98, 1233–1240. 24025626 10.3945/ajcn.113.061549

[13] Torsvik IK , Ueland PM , Markestad T , et al. (2015) Motor development related to duration of exclusive breastfeeding, B vitamin status and B_12_ supplementation in infants with a birth weight between 2000–3000 g, results from a randomized intervention trial. BMC Pediatr 15, 218. 26678525 10.1186/s12887-015-0533-2 PMC4683944

[14] Kvestad I , Taneja S , Kumar T , et al. (2015) Vitamin B_12_ and folic acid improve gross motor and problem-solving skills in young North Indian children: a randomized placebo-controlled trial. PLOS ONE 10, e0129915. 26098427 10.1371/journal.pone.0129915 PMC4476750

[15] Strand TA , Ulak M , Hysing M , et al. (2020) Effects of vitamin B_12_ supplementation on neurodevelopment and growth in Nepalese infants: a randomized controlled trial. PLoS Med 17, e1003430. 33259482 10.1371/journal.pmed.1003430 PMC7707571

[16] Institute of Medicine Standing Committee on the Scientific Evaluation of Dietary Reference I, OBV its Panel on Folate & Choline (1998) The national academies collection: reports funded by national institutes of health. In Dietary Reference Intakes for Thiamin, Riboflavin, Niacin, Vitamin B(6), Folate, Vitamin B(12), Pantothenic Acid, Biotin, and Choline. Washington, DC: National Academies Press (US), National Academy of Sciences.

[17] Brito NH , Fifer WP , Amso D , et al. (2019) Beyond the Bayley: neurocognitive assessments of development during infancy and toddlerhood. Dev Neuropsychol 44, 220–247. 30616391 10.1080/87565641.2018.1564310 PMC6399032

[18] Rubio-Codina M & Grantham-McGregor S (2020) Predictive validity in middle childhood of short tests of early childhood development used in large scale studies compared to the Bayley-III, the family care indicators, height-for-age, and stunting: a longitudinal study in Bogota, Colombia. PLOS ONE 15, e0231317. 32348359 10.1371/journal.pone.0231317 PMC7190101

[19] Bayley N (2006) Manual for the Bayley Scales of Infant and Toddler Development. San Antonio, TX: NCS Pearson.

[20] Murray-Kolb LE , Rasmussen ZA , Scharf RJ , et al. (2014) The MAL-ED cohort study: methods and lessons learned when assessing early child development and caregiving mediators in infants and young children in 8 low- and middle-income countries. Clin Infect Dis 59, Suppl. 4, S261–S272. 25305296 10.1093/cid/ciu437 PMC4204608

[21] Ranjitkar S , Kvestad I , Strand TA , et al. (2018) Acceptability and reliability of the Bayley scales of infant and toddler development-III among children in Bhaktapur, Nepal. Front Psychol 9, 1265. 30087639 10.3389/fpsyg.2018.01265 PMC6066572

[22] Wechsler D (2012) Wechsler Preschool and Primary Scale of Intelligence, 4th ed. San Antonio, TX: The Psychological Corporation.

[23] Colom R (2004) Intelligence assessment. In Encyclopedia of Applied Psychology, pp. 307–314 [ C Spielberger , editor]. San Diego: Academic Press.

[24] Korkman M , Kirk U & Kemp S (1998) NEPSY: A Developmental Neuropsychological Assessment Manual. San Antonio, TX: The Psychological Corporation.

[25] Brooks BL , Sherman EM & Strauss E (2009) NEPSY-II: a developmental neuropsychological assessment. Child Neuropsychol 16, 80–101.

[26] Strand TA , Ulak M , Chandyo RK , et al. (2017) The effect of vitamin B_12_ supplementation in Nepalese infants on growth and development: study protocol for a randomized controlled trial. Trials 18, 187. 28431557 10.1186/s13063-017-1937-0 PMC5399862

[27] StataCorp. (2017) Stata Statistical Software: Release 15. College Station, TX: StataCorp. LLC.

[28] Mercedes DO (2006) WHO child growth standards based on length/height, weight and age. Acta Paediatr 95, 76–85. 10.1111/j.1651-2227.2006.tb02378.x 16817681

[29] Chandyo RK , Ulak M , Kvestad I , et al. (2018) Cobalamin and folate status among breastfed infants in Bhaktapur, Nepal. Nutrients 10, 639. 29783689 10.3390/nu10050639 PMC5986518

[30] Kvestad I , Taneja S , Upadhyay RP , et al. (2020) Vitamin B_12_, folate, and cognition in 6- to 9-year-olds: a randomized controlled trial. Pediatrics 145, e20192316. 32019814 10.1542/peds.2019-2316

