# Supplementary material for: The effect of infant vitamin B12 supplementation on neurodevelopment: a follow-up of a randomised placebo-controlled trial in Nepal
Source: Br J Nutr. 2022 Feb 14;129(1):41–8. doi: 10.1017/S0007114522000071 (PMC9816651; doi:10.1017/S0007114522000071)
Supplement: Supplementary file 1 [file S0007114522000071sup001.pdf]

Composition of micronutrients in supplement paste of vitamin B12 and placebo:

	Treatment product with vitamin B12				Placebo without vitamin B12		
**Vitamins Per**	100 g	20 g	claim 20 g sachet		100 g	20 g	claim 20 g sachet
Vitamin A (µg)	2000 - 3000	400 - 600	400		2000 - 3000	400 - 600	400
Vitamin C (mg)	150 - 375	30 - 75	40		150 - 375	30 - 75	40
Vitamin B1 (mg)	1.5 - 3.0	0.3 - 0.6	0.3		1.5 - 3.0	0.3 - 0.6	0.3
Vitamin B2 (mg)	2.0 - 2.6	0.4 -0.52	0.4		2.0 - 2.6	0.4 - 0.52	0.4
Vitamin B6 (mg)	1.4 - 2.0	0.28 - 0.4	0.28		1.4 - 2.0	0.28 -0.4	0.28
Vitamin B12 (µg)	10 - 20	2.0 - 4.0	2,0		-	-	-
Niacin (mg)	20 - 28	4.0 - 5.6	4.0		20 - 28	4.0 - 5.6	4.0
Ca-D-Pantothenate (mg)	8.0 - 14.0	1.6 - 2.8	1.8		8.0 - 14.0	1.6 - 2.8	1.8
Folic acid (µg)	400 - 550	80 - 110	80		400 - 550	80 - 110	80
**Minerals per**							
Calcium (mg)	450 - 650	90 - 130	104		450 - 650	90 - 130	104
Phosphor (mg)	400 - 500	80 - 100	85		400 - 500	80 - 100	85
Potassium (mg)	700 - 850	140 - 170	146		700 - 850	140 - 170	146
Magnesium (mg)	70 - 90	14 - 18	15.2		70 - 90	14 - 18	15.2
Iron (mg)	40 - 50	8.0 - 10	8.6		40 - 50	8.0 - 10	8.6
Zinc (mg)	18 - 20	3.6 - 4.4	3,8		18 - 20	3.6 - 4.4	3,8
Copper (mg)	0.9 - 1.1	0.18 - 0.22	0.19		0.9 - 1.1	0.18 - 0.22	0.19
Iodine (µg)	400 - 500	80 - 100	84.5		400 - 500	80 - 100	84.5
Selenium (µg)	45 - 55	9.0 - 11.0	10		45 - 55	9.0 - 11.0	10
Manganese (mg)	0.36 - 0.45	0,07 - 0.10	0.08		0.36 - 0.45	0,07 - 0.10	0.08
